# Associations of select neuropathologies with cardiovascular risk factors in a diverse cohort with autopsy‐confirmed Alzheimer disease

**DOI:** 10.1002/alz70855_103850

**Published:** 2025-12-24

**Authors:** Hsin‐Pei Wang, Naomi Saito, Laurel Beckett, Lawrence S. Honig, Charles S. DeCarli, Robert A. Rissman, Andrew F. Teich, Dan M. Mungas, Lee‐Way Jin, Brittany N Dugger

**Affiliations:** ^1^ University of California, Davis, Sacramento, CA, USA; ^2^ University of California, Davis, Davis, CA, USA; ^3^ Taub Institute for Research on Alzheimer's Disease and the Aging Brain, Vagelos College of Physicians and Surgeons, Columbia University, New York, NY, USA; ^4^ UC Davis, Davis, CA, USA; ^5^ University of California, San Diego, La Jolla, CA, USA; ^6^ Taub Institute for Research on Alzheimer's Disease and the Aging Brain, New York, NY, USA

## Abstract

**Background:**

Cardiovascular risk factors have been implicated in the progression of Alzheimer disease (AD) and its neuropathological hallmarks, yet their specific contributions to regional brain pathology in diverse populations remain understudied.

**Method:**

In this cohort study (*n* = 276), we examined the relationship between three‐level categorizations (absent, active/recent, inactive/remote) of diabetes, hypertension, and hypercholesterolemia, and neuropathologies in a diverse group of Hispanic and non‐Hispanic White decedents with pathologically confirmed Intermediate/High AD from three Alzheimer's Disease Research Centers. Semi‐quantitative assessments for regional arteriolosclerosis, cerebral amyloid angiopathy (CAA) density, core plaques (CPs), diffuse plaques (DPs), and neuropil threads (NTs) were done adapting scales from established CERAD criteria.

**Result:**

Active diabetes was associated with increased density of frontal NTs (*p* <0.01), while active hypercholesterolemia correlated with greater density of CAA in temporal cortex (*p* = 0.04) and posterior hippocampus (*p* = 0.03), as well as temporal CPs (*p* = 0.03), as determined by Kruskal‐Wallis test. No significant associations were observed between hypertension status and neuropathology. Ordinal logistic regression adjusting for cardiovascular risk factors, ethnicity, sex, age of death, and center confirmed these associations except for CPs in the temporal cortex.

**Conclusion:**

This study underscores the importance of incorporating diverse cohorts in AD research to ensure broader generalizability and shows links between cardiovascular risk factors and AD‐related neuropathologies, advancing precision medicine approaches for dementia.

